# Low expression of Aldo–keto reductase 1B10 is a novel independent prognostic indicator for nasopharyngeal carcinoma

**DOI:** 10.1186/s13578-016-0082-x

**Published:** 2016-03-05

**Authors:** Yuanwei Guo, Weihao Luo, Zheng Hu, Jia Li, Xiaojie Li, Huiqiu Cao, Jun Li, Bo Wen, Jian Zhang, Hao Cheng, Wangyuan Guo, Tan Tan, Dixian Luo

**Affiliations:** Translational Medicine Institute, National and Local Joint Engineering Laboratory for High-through Molecular Diagnosis Technology, Collaborative Research Center for Post-doctoral Mobile Stations of Central South University, Affiliated The First People’s Hospital of Chenzhou, University of South China, 432000 Chenzhou, People’s Republic of China; Center for Clinical Pathology, Affiliated The First People’s Hospital of Chenzhou, University of South China, 432000 Chenzhou, People’s Republic of China; E.N.T. Department, The First People’s Hospital of Chenzhou, 432000 Chenzhou, People’s Republic of China; Department of Clinical Pharmacology, Xiangya Hospital and Institute of Clinical Pharmacology, Central South University and Hunan Key Laboratory of Pharmacogenetics, 410078 Changsha, Hunan People’s Republic of China

**Keywords:** AKR1B10, Nasopharyngeal carcinoma, Metastasis, Prognostic marker, Low expression

## Abstract

**Background:**

Nasopharyngeal carcinoma (NPC) is one of the most common human head and neck cancers with high incidence in Southern China, Southeast Asia and North Africa. Because of its nonspecific symptoms, the early diagnosis of NPC is very difficult. The 5-year survival rate is not ideal in spite of great innovations in radiation and chemotherapy treatments. Highly sensitive and specific prognostic biomarkers are eager for NPC clinical diagnosis. To find specific target molecules is very important for individualized treatment. Aldo–keto reductase B10 (AKR1B10) is closely related to tumorigenesis and tumor development, and however, its expression level in NPC tissues is not clear.

**Results:**

AKR1B10 expression levels were validated in benign, para-cancerous nasopharyngeal and NPC tissues by immunohistochemical evaluation. AKR1B10 was positively expressed in 42 (82.4 %) of 51 benign specimens, and 235 (98.7 %) of 238 para-carcinoma specimens. This percentage was significantly higher than 44.5 % (133/299) in nasopharyngeal carcinoma tissue (*p* < 0.01). AKR1B10 mRNA quantitative levels detected by real-time quantitative RT-PCR in 90 NPC tissue samples (0.10 ± 0.21) were significantly lower than that in 15 benign tissue samples (1.03 ± 1.12) (*p* < 0.01). AKR1B10 expression levels in NPC were correlated negatively with T-classification, lymph node metastasis (*p* < 0.05). We established nasopharyngeal cancer monoclonal cells CNE-2/AKR1B10 with AKR1B10 stable expression and CNE-2/vector cells without AKR1B10 expression by using a modified lentivirus-mediated method, and found that AKR1B10 inhibited the proliferation of CNE-2/AKR1B10 cells by using MTT assay and flow cytometry, and cell migration by in vitro scratch test.

**Conclusion:**

Taken together, our data suggest that low expression of AKR1B10 is an independent prognostic indicator in nasopharyngeal carcinoma, and that AKR1B10 may be involved in regulating the proliferation and migration of nasopharyngeal cancer cells.

## Background

Nasopharyngeal carcinoma (NPC) is one of the most common human head and neck cancers. Its development has been related to Epstein-Barr virus (EBV) infection, hereditary and environmental factors, and behavioral habits. Incidence of NPC is high in Southern China, Southeast Asia and North Africa [1–3], and the incidence is constantly increasing as air pollution and food contamination have been deteriorating in recent years [4]. Due to its nonspecific symptoms in the early stage, early diagnosis is very difficult, which has caused serious global health problems.

At present, the pathogenesis of NPC is not very clear [5], and the treatment options are limited in the absence of effective and specific therapeutic drugs. NPC is a heterogeneous group of diseases, with substantial differences in pathological types, degrees of tumor differentiation, clinical manifestations, physical fitness, and sensitivity to radiation and chemotherapy in different patients. Therefore, the outcome of the therapeutic interventions is not ideal, and the 5-year survival rate is still about 50 % in spite of great innovations in radiation and chemotherapy treatments [6, 7]. At present, NPC is mainly diagnosed by imaging exams, but the high cost of the tests, the inaccuracy of evaluation of treatment efficacy and the poor sensitivity in diagnosing metastasis have limited their clinical use [8]. High expression of matrix metalloproteinase 1 (MMP-1) and lower expression of tissue factor pathway inhibitory factor 2 (TFPI-2) are closely related to neck lymph node metastasis of NPC. For this reason, they are used as a biological indicator in the assessment of NPC metastasis in advanced stage tumors [9, 10]. However, highly sensitive and specific prognostic biomarkers are not available for NPC clinical diagnosis. It is very important to screen out novel specific molecular indicators of NPC for early diagnosis and individualized treatment.

Aldo-keto reductase family 1 member B10 (AKR1B10), also called as aldose reductase like protein-1 (ARL-1), belongs to the aldehyde ketone reductase (AKRs) family [11]. Its coding gene is in the q33 region of chromosome 7, encoding a protein composed of 316 amino acids, which was isolated from hepatocellular carcinoma in 1998 [12]. AKR1B10 not only regulates the balance of retinoic acid and lipid metabolism, but also participates in cell survival through cytotoxic carbonyl detoxification. AKR1B10 is expressed at different levels in different human cancer cells, affecting the growth and survival of tumors, and its activity is closely related to tumor development [13–15]. AKR1B10 is highly expressed in normal gastric intestinal epithelial tissue, whereas its expression is low or absent in other normal tissues. AKR1B10 is highly expressed in variety of solid tumors such as liver cancer, non-small cell lung cancer, breast cancer, and pancreatic cancer [16–23]. However AKR1B10 was found to be expressed lowly or negatively in digestive tract tumors like as esophageal, gastric and colon cancer [24–27]. Therefore, AKR1B10 is a potential tumor biomarker and therapeutic target.

AKR1B10 expression in NPC is not clear. In this study, we identified AKR1B10 expression level in NPC, and the relationship between the expression level and clinicopathological parameters. The study results suggest that AKR1B10 may be a novel prognostic factor for human NPC.

## Results

### Lower expression of AKR1B10 in NPC

**Fig. 1:**
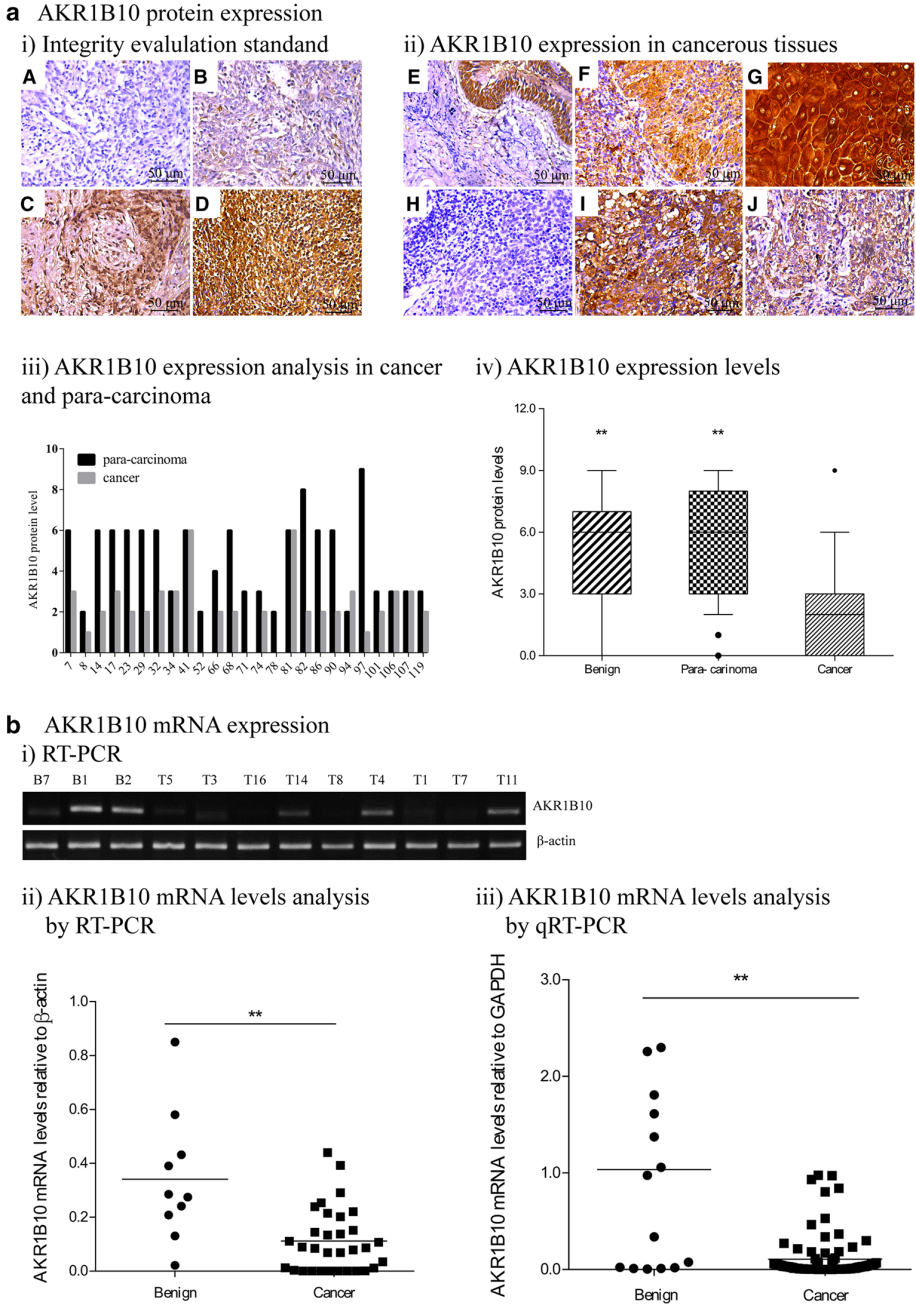
AKR1B10 expression in NPC tissues. **a** AKR1B10 protein expression was detected in 299 NPC specimens, 238 para-carcinoma specimens and 51 benign specimens by immunochemistry. *i* Immunohistochemical AKR1B10 staining was evaluated by scoring the staining intensity from 0 to 3+ : 0 for no staining (*A*), 1+ for weak immunoreactivity (*B*), 2+ for moderate immunoreactivity (*C*), 3+ for strong immunoreactivity (*D*). *ii* According to the standard, AKR1B10 expression was evaluated in: *E* inflammatory hyperplasia; *F* adenoid hypertrophy; *G* para-carcinoma; *H* undifferentiated nasopharyngeal carcinoma; *I* differentiated nasopharyngeal carcinoma; *J* mandibular lymphatic metastasis. *iii* AKR1B10 protein levels (immunohistochemical staining scoring) were analyzed in 25 randomly selected paired cases of NPC and para-carcinoma. *iv* AKR1B10 expression levels relative to β-actin were compared in 51 benign, 238 para-carcinoma and 299 NPC tissue specimens (**p < 0.01). **b** *i* AKR1B10 mRNA expression detected by RT-PCR in some representative specimens was shown. B, Benign and T, Tumor. *ii* AKR1B10 mRNA levels relative to β-actin examined by RT-PCR were compared in 32 NPC and 10 benign tissue specimens (**p < 0.01). *iii* AKR1B10 mRNA levels relative to GAPDH examined by qRT-PCR were compared in 90 NPC and 15 benign tissue specimens (**p < 0.01)

AKR1B10 protein levels were examined in 51 benign specimens, 238 para-carcinoma specimens and 299 nasopharyngeal cancer specimens by immunochemistry. Yellow–brown granules corresponding to AKR1B10 were detected mainly in the cytoplasm and occasionally in the nucleus. AKR1B10 expression levels were scored for staining intensity from 0 to 3+ (Fig. 1a-i). AKR1B10 expression was detected in nasopharyngeal benign and para-cancerous tissues, adenoids, inflammatory hyperplasia, differentiated nasopharyngeal carcinoma, undifferentiated nasopharyngeal carcinoma, and lymphatic metastases (Fig. 1a-ii). The immunohistochemical evaluation histogram of 25 randomly selected cases showed that AKR1B10 protein expression levels (evaluation scores) in the cancerous tissue were lower than those in para-carcinoma tissue in 19 cases, and the expression levels were similar in between cancerous and para-carcinoma tissues in only five cases, and the expression level in para-carcinoma tissue was higher than that in cancerous tissue in only one case (Fig. 1a-iii). The percentage of AKR1B10-positive specimens was 44.5 % (133/299) in the nasopharyngeal cancer tissue compared with 82.4 % (42/51) in benign specimens (*p* < 0.01) and 98.7 % (235/238) in para-carcinoma specimens (*p* < 0.01) (Table 1). The evaluation scores of AKR1B10 expression in carcerous tissue was 1.91 ± 1.76, significantly lower than 5.52 ± 2.5 in benign specimens and 5.20 ± 2.89 in para-carinoma specimens(p < 0.01) (Fig. 1a-iv).

**Table 1:** Expression of AKR1B10 in benign, para-carcinoma and cancer tissues

	AKR1B10 expression		Subtotal	χ^2^	*p* value
	Positive N (%)	Negative N (%)			
Benign	42 (82.4)	9 (17.6)	51		
Para-carcinoma	235 (98.7)	3 (1.3)	238		
NPC	133 (44.5)	166 (55.5)	299	227.507	<0.001
Total	410 (69.7)	178 (30.3)	588		

To investigate AKR1B10 mRNA expression in NPC, we extracted from total mRNA from 32 fresh NPC and 10 benign tissue specimens, and AKR1B10 mRNA levels were examined by RT-PCR. Human β-actin served as an internal control, the gray-scale comparison between AKR1B10 and β-actin was used to quantify the bands of RT-PCR. As shown in Fig. 1b, we observed significantly lower AKR1B10 mRNA levels in NPC tissues (0.11 ± 0.02) than that in benign tissues(0.34 ± 0.08) (*p* < 0.01). To further investigate quantitative expression levels of AKR1B10 mRNA in NPC, we collected 90 NPC specimens and 15 benign specimens, which were subjected to ARK1B10 mRNA quantitative detection by using real-time qRT-PCR. Human GAPDH served as an internal control, and data were analyzed using the 2^∆CT^ method, where ∆CT = Ct_AKR1B10_ − Ct_GAPDH_. We observed that AKR1B10 mRNA quantitative levels were lower in NPC (0.10 ± 0.21) than that in benign specimens (1.03 ± 1.12) (*p* < 0.01), which was consist with the RT-PCR results.

### Correlationship between AKR1B10 expression and clinicopathological features in NPC

200 NPC tissue specimens with detailed clinical medical records were subjected to detecting AKR1B10 protein expression levels by immunochemistry, and AKR1B10 were highly expressed in 15 cases (evaluation score of AKR1B10 expression = 3), moderately expressed in 33 cases (score = 2), weakly expressed in 73 cases (score = 1), and negatively expressed in 79 cases (score = 0). We statistically analyzed correlationship between AKR1B10 expression levels evaluated by immunochemistry methods and clinicopathological features, such as patient age, tumor type (differentiation), neck masses, tumor size (T classification), node metastasis (N classification), distant metastasis, TNM clinical stage (Table 2). The frequency of AKR1B10 positivity was significantly higher in patients with differentiated NPC than that in patients with undifferentiated NPC (71.2 %, 89/125 vs 42.7 % 32/75, *p* < 0.01). Furthermore, AKR1B10 expression levels in NPC were negatively correlated with tumor size (r = −0.399, *p* < 0.001) and lymph node metastasis (r = −0.326, *p* < 0.001).

**Table 2:** Correlation between AKR1B10 expression levels and the clinicopathologic characteristics of NPC patients

Variables	AKR1B10 expression levels				Sub-total	Fisher’s exact tests		Correlation analysis	
	3	2	1	0		Values	*p* values	r values	*p* values
Subtotal	15	33	73	79	200				
Age (years)									
≤45	9	10	31	29	79	4.235	0.239	0.046	0.519
>45	6	23	42	50	121				
Tumor type									
Undifferentiation	6	10	16	43	75	18.008	<0.001	0.212	0.003
Differentiation	9	23	57	36	125				
Neck masses (cm)									
0	8	15	26	33	82	2.765	0.848	0.047	0.510
>0, ≤3	4	10	15	13	42				
>3	3	8	32	33	76				
T-classification									
T1/T2	10	21	33	18	82	36.119	<0.001	-0.399	<0.001
T3/T4	5	12	40	61	118				
N classification									
N0/N1	12	23	37	26	98	19.746	<0.001	-0.311	<0.001
N2/N3	3	10	36	53	102				
Distant metastasis									
Yes	6	6	5	18	35	12.776	0.004	0.011	0.892
No	9	27	68	61	165				
TNM clinical stage									
I–II	12	30	67	66	175	3.569	0.302	0.059	0.420
III–IV	3	3	6	13	25				

### Effect of AKR1B10 on the proliferation of NPC cells

**Fig. 2:**
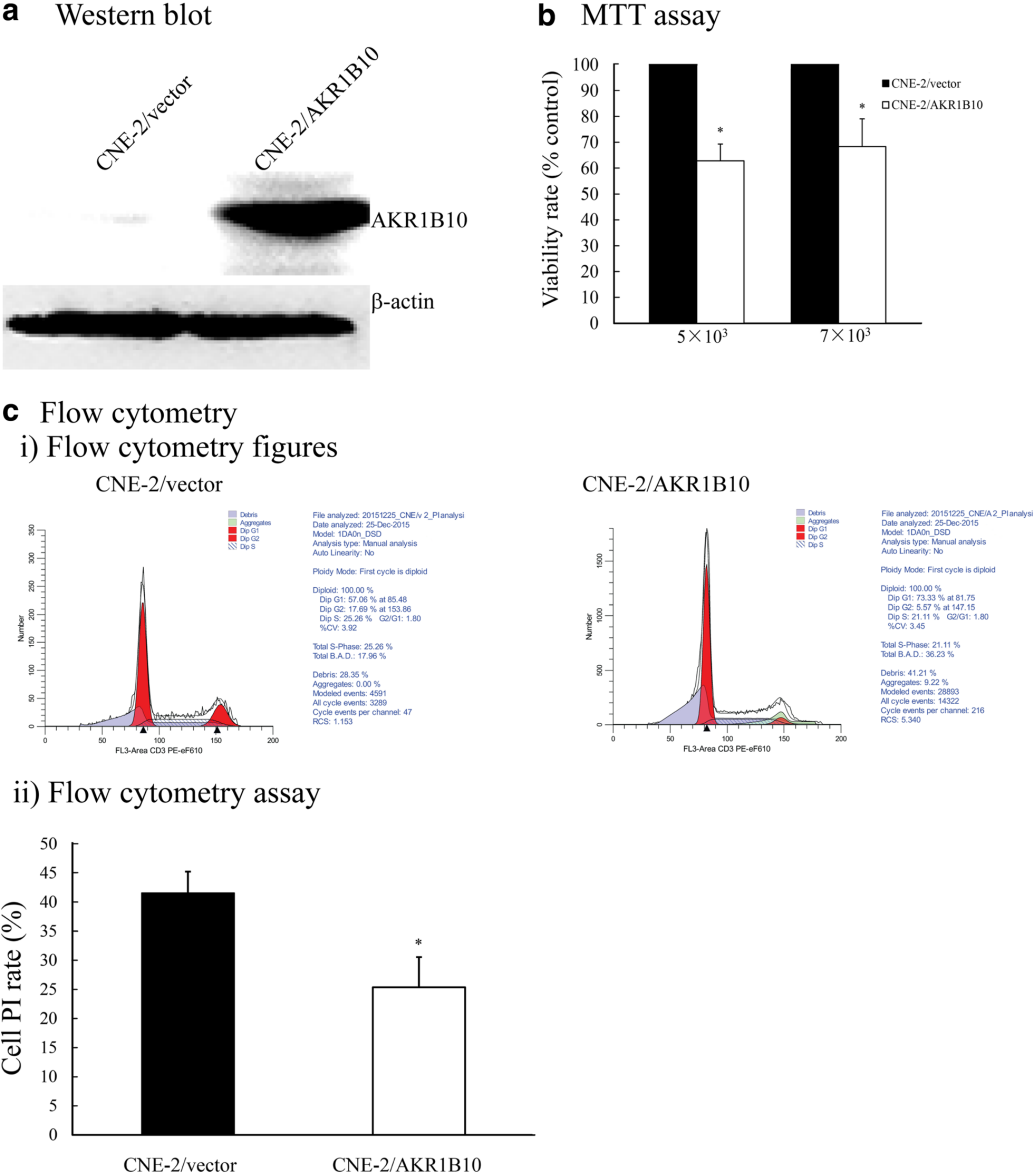
Effect of AKR1B10 on the proliferation of NPC cells. **a** AKR1B10 protein expression was assayed in CNE-2/AKR1B10 and CNE-2/vector cells by western blot. **b** 5 × 10^3^ or 7 × 10^3^ CNE-2/vector and CNE-2/AKR1B10 cells per well were seeded into 96-well plates and cultured for 72 h. Cell proliferation was analyzed by MTT assay (*p < 0.05). **c** Cell cycle distribution was detected by propidium iodide staining and flow cytometry. *i* Representative cell cycle flow cytometry images of CNE-2/vector and CNE-2/AKR1B10 cells. *ii* Cell proliferation index (PI) was calculated according to the equation: PI = (S % + G2/M %)/(G0/G1 % + S % + G2/M %) × 100 %, and the PI of CNE-2/vector cells was higher than that of CNE-2/AKR1B10 cells (*p < 0.05). All the experiments were repeated three times at least

Given the negative correlation of AKR1B10 expression levels with tumor size, we hypothesized that AKR1B10 could influence cell proliferation of NPC cells. To identify this hypothesis, we established CNE-2/AKR1B10 monoclonal cells with AKR1B10 stable expression, and CNE-2/vector cells without AKR1B10 expression by using a modified lentivirus-mediated method, and examined over-expression of AKR1B10 in CNE-2/AKR1B10 cells and negative expression of AKR1B10 in CNE-2/vector cells by western blot (Fig. 2a). The proliferation of CNE-2/vector cells and CNE-2/AKR1B10 cells were measured by MTT assay and flow cytometry. As shown in Fig. 2b, the viability rate of 5 × 10^3^ or 7 × 10^3^ CNE-2/AKR1B10 cells assayed by MTT experiments were respectively 62.84 ± 6.50 and 68.37 ± 10.73 %, related to that of CNE-2/vector cells (*p* < 0.05). The proliferation index (PI) was significantly lower in CNE-2/AKR1B10 (25.37 ± 5.18 %) than that in CNE-2/vector cells (41.56 ± 3.67 %) (*p* < 0.05), as shown in Fig. 2c.

### Effect of AKR1B10 on the migration of NPC cells

**Fig. 3:**
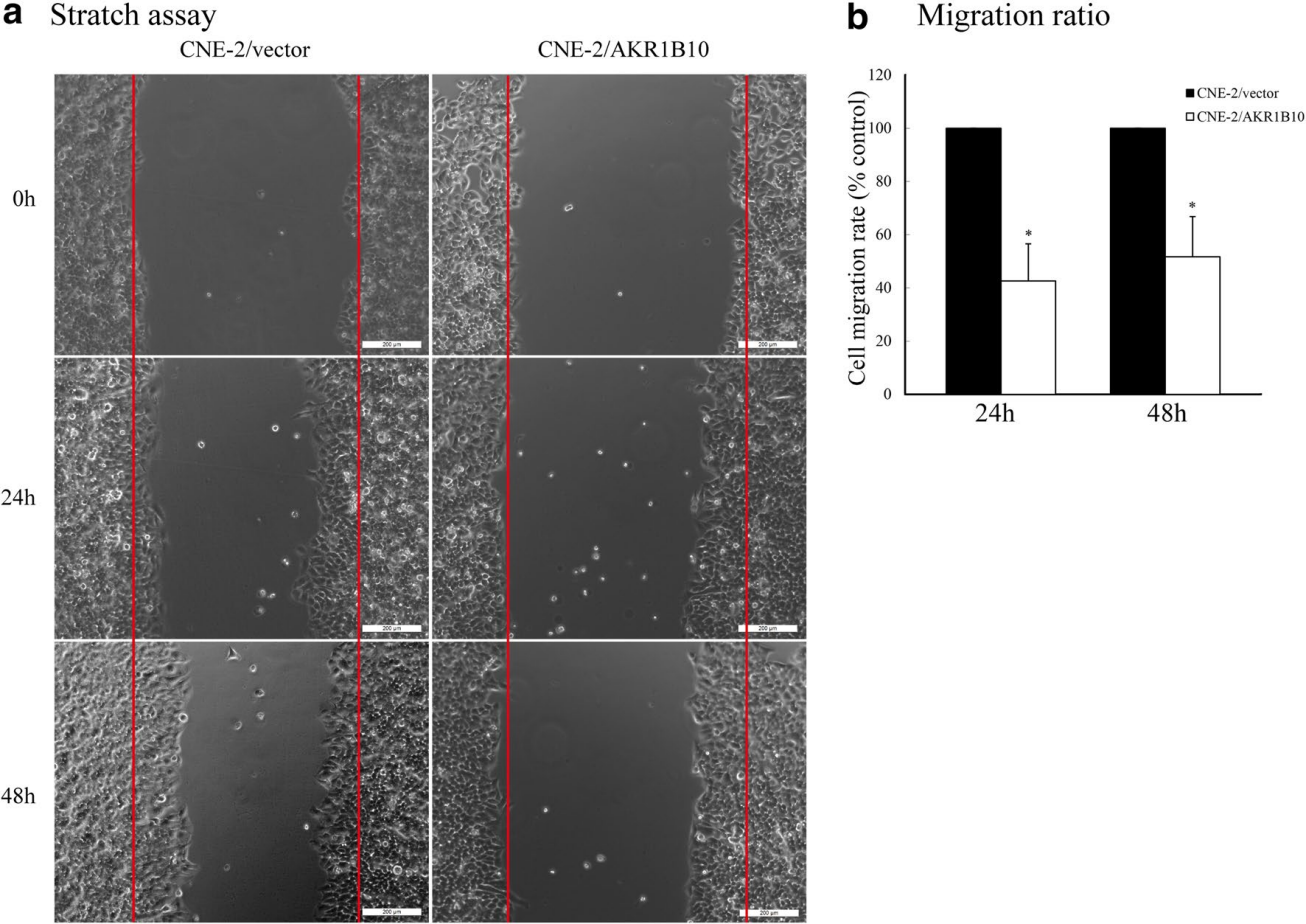
Effect of AKR1B10 on the migration of NPC cells. **a** *In vitro* scratch assay. The same numbers of CNE-2/vector and CNE-2/AKR1B10 cells were cultured in 6-well plates. The bottoms of the plates were scratched when the cells were cultured to confluency. The images were captured at 0, 24 and 48 h. **b** Histogram of cell migration ratio (MR) calculated by using the equation: MR % = [(Area at T_0_ − Area at T_t_)/Area at T_0_] × 100 % (t = 24 h or t = 48 h). The experiments were repeated three times at least (*p < 0.05)

According to the negative correlation of AKR1B10 expression levels and tumor node metastasis, we thought that AKR1B10 could have influenced cell migration of NPC cells. The migration experiments of CNE-2/vector cells and CNE-2/AKR1B10 cells were done by in vitro scratch assay. As shown in Fig. 3a and 3b, the scratch area was measured by *Image*-*pro plus*, and the migration rates (MR) were calculated according to the equation: MR % = [(Area at T_0_ − Area at T_t_)/Area at T_0_] × 100 %, where T_t_ is the number of hours post-injury and T_0_ is the time of injury. As shown in Fig. 3, the migration rates of CNE-2/AKR1B10 cells at 24 h or 48 h were respectively 42.62 ± 13.99 and 51.7 ± 15.12 %, relative to that of CNE-2/vector.

## Discussion

NPC is a heterogeneous group of diseases characterized by different biological behaviors. To better manage patients with NPC, novel tumor-specific markers are required for diagnosis, targeted therapy, and prognosis prediction. In this study, we report that AKR1B10 expression is down-regulated in NPC, and AKR1B10 may represent a new clinicopathological biomarker and prognostic factor for this disease.

AKR1B10 has been reported to be overexpressed in many non-digestive tract solid tumors such as non-small cell lung cancer in smokers, breast cancer, pancreatic cancer and hepatocellular carcinoma [16, 18, 21, 28–30]. Recent clinicopathological studies demonstate that AKR1B10 may be a valuable biomarker of differentiation and proliferation in liver tumors, and may also play a role in early phases of hepatocarcinogenesis [28, 31]. However, AKR1B10 expression is downregulated in gastrointestinal cancers. It is very interesting that AKR1B10 expression shows an opposite pattern in digestive tract cancers compared with cancers arising outside of the digestive tracts. In the present study, we have found that both AKR1B10 protein and mRNA levels were reduced in most of NPC tumors compared with para-carcinoma and benign tissues, which further suggests that AKR1B10 expression is down-regulated in the digestive tract cancers. He et al. [32] also investigated that AKR1B10 expression levels in NPC tissues are lower than that in benign tissues, which is consistent to our results. However, in their studies, AKR1B10 expression levels are higher in NPC tissues than that in normal nasopharyngeal tissues of healthy people [32], which was not observed in our studies because it was too difficult to obtain normal nasopharyngeal tissues from healthy persons. Therefore, we only compare AKR1B10 expression in NPC tissues with that in para-carcinoma tissues in same NPC patients or with that in benign tissues in other patients.

These digestive tract organs are directly exposed to luminal carbonyl toxins from the food, water and air or from local production by the microbiome [33]. Cao et al. [34] published that AKR1B10 is an important protector in the gastrointestinal epithelium. AKR1B10 plays important roles in detoxification of cytotoxic carbonyl compounds, and reduction of various aliphatic and aromatic α, β-unsaturated aldeketones to the respective alcohols [35]. The carbonyls were also produced during cell metabolism via the lipid peroxidation, which induces protein dysfunction, DNA damage and oncogenesis [35, 36]. By efficiently detoxifying cellular carbonyls and their glutathione conjugates by reduction of reactive carbonyl groups into less active hydroxyls, AKR1B10 may protect host cells from carbonyl-induced lesions [34–37]. It was reported that N,N′-Dinitrosopiperazine (DNP), as substrates of AKR1B10 enzyme, increased the expression of AKR1B10 in nasopharyngeal carcinoma 6-10B cells [38]. Though AKR1B10 is not only predominantly expressed in the distal gastrointestinal tract, but also in proximal nasopharynx, some nasopharyngeal cells with low or negative expression of AKR1B10 in some NPC patients might undergo carcinogenesis and eventually grow into tumors due to loss of efficient detoxification of AKR1B10, or excessive cytotoxic carbonyls.

Recent clinicopathological studies demonstrate that AKR1B10 expression in hepatocellular carcinomas differs depending on the disease stage. AKR1B10 is significantly overexpressed in lower tumor stages with underlying cirrhosis or viral hepatitis, whereas it is down-regulated in advanced tumor stages [28]. Therefore, AKR1B10 may be a valuable biomarker in many tumors. Our results showed that AKR1B10 expression levels are negatively correlated with tumor types, neck masses, and lymph node metastasis of NPC, and positively correlated with NPC differentiation. The positive correlation was also observed in Dr. He’s study [32]. However, the negative correlations in NPC are unlike in solid cancers outside of the digestive tract system. The cell proliferation and migration assays further confirmed the negative correlation, showing that AKR1B10 inhibits cell proliferation and migration of NPC. However, In non-digestive tract solid tumors such as lung cancer, breast cancer, and hepatocellular carcinoma, AKR1B10 are over-expressed and stimulates cell proliferation and migration by promoting lipid synthesis, activating the signaling molecule retinoic acid and transcription factor Nfr-2 that regulate cell proliferation and differentiation [39]. However, the mechanism on AKR1B10 suppressing cell proliferation and migration is not clear, which we are studying.

Therefore, we conclude that AKR1B10 may be used as a prognostic marker for nasopharyngeal carcinoma. The cause of opposite pattern of expression in cancers inside or outside the digestive tracts might be related to unknown functions of AKR1B10 in different tissues. In summary, AKR1B10 is negatively correlated with differentiation, tumor growth and lymph node metastasis by inhibiting cell proliferation and migration of NPC, and therefore, AKR1B10 may be a valuable clinicopathological and prognostic indicator in patients with NPC.

## Conclusion

Our data demonstrates that AKR1B10 may be involved in regulating the proliferation and migration of nasopharyngeal cancer cells, and AKR1B10 expression levels are negative correlated with lymph node metastasis of nasopharyngeal carcinoma. Therefore low expression of AKR1B10 is an independent prognostic indicator in NPC.

## Methods

### Tissues and clinical data

With an IRB protocol (ky2013005) approved by Ethics Committee of the First People’s Hospital of Chenzhou, we procured paraffin-embedded formalin-fixed specimens including benign specimens (n = 51), nasopharyngeal cancer specimens (n = 299) and para-carcinoma specimens (n = 238) collected and identified were identified from the archives of Department of Pathology in the First People’s Hospital of Chenzhou between 2008 and 2015. The samples were used to detect AKR1B10 expression by immunochemical assay. Freshly frozen tumor tissues (n = 90) and benign tissues (n = 15) were obtained from Department of E.M.T. in the First People’s Hospital of Chenzhou, and were used for AKR1B10 mRNA analysis by RT-PCR and qRT-PCR. All the samples with pathological diagnosis were confirmed by the professors in Chenzhou Pathology Center in the First People’s Hospital of Chenzhou.

### Immunohistochemistry method

For immunohistochemical analysis, paraffin-embedded tissue sections were deparaffinized in xylene, washed with ethanol, rehydrated, and then incubated with fresh 3 % (v/v) H_2_O_2_ for 10 min for eliminating endogenous peroxidase activity. After being rinsed with distilled water, sections were washed three times with phosphate-buffered saline (PBS) for 5 min each time, and then incubated with 10 % (v/v) normal goat serum (diluted in PBS) at room temperature for 20 min. The sections were then incubated with rabbit anti-AKR1B10 antibodies for 1.5 h at 37 °C, followed by incubation with HRP-labeled secondary antibody at 37 °C for 30 min. Subsequently, the sections were stained with 3,3-diaminobenzidine (DAB) for 5–10 min and counterstained with hematoxylin.

All slides were observed under a Leica light microscope DM4 B (Leica Corporation, Germany) at high magnification and five microscopic fields were randomly selected for immunochemical evaluation. AKR1B10 expression levels were scored on staining intensities from 0 to 3+ (0 for no staining, 1+ for weak immunoreactivity, 2+ for moderate immunoreactivity and 3+ for strong immunoreactivity). The percentage of cells with positive AKR1B10 staining within the cancerous region of a section were scored as follows: 0 for <5 % positive cells, 1+ for 5‒10 %, 2+ for 11–50 % and 3+ for 51‒100 %. The percentages within para-cancerous region of the same section were scored according to the same evaluation standard. The intensity and proportion scores were then multiplied to obtain a composite score. The values of the composite score ranged between 0 and 9, and we defined a score of <2 as negative, of 2‒3 as weakly positive, of 4‒5 as moderately positive, and of ≥6 as strongly positive.

### RNA preparation, RT-PCR and real-time fluorescence quantative RT-PCR (qRT-PCR)

Total RNA was extracted using Trizol^®^ reagent (Invitrogen, CA) and quantitated at OD_260_ with a multiscan spectrometer (Thermo Varioskan Flash, Wilmington, DE). The RNA integrity was verified by gel electrophoresis. RNA was treated with RNase-free DNase 1, and the first-strand cDNA was synthesized from 1 µg of total RNA with oligo-dT primers and GoSript^®^ retrotranscriptase, following the manufacturer’s protocol (Promega, USA). The PCR reactions were performed as follows: initial denaturation at 94 °C for 5 min, implification (94 °C for 30 s, 58 °C for 45 s, 72 °C for 30 s, and 32 cycles), and final extension at 72 °C for 10 min. β-actin mRNA in each sample was run in parallel for an internal control. Primers for AKR1B10 are 5′-CTG GAT CCG GCA AGA TTA AGG AGA T (forward) and 5′-GAC TGC GGC CGC GAT ATC CAC CAG G (reverse), and primers for β-actin are 5′-ATC ATT GCT CCT CCT GAG CGC (forward) and 5′-TGA ACT TTG GGG GAT GCT CGC (reverse). The PCR products were run in agarose-gel, and the gray values of AKR1B10 and β-actin were scanned by Alpha View SA 3.30 (Cell Biosciences, USA) and semi-quantitative expression level of AKR1B10 was calculated relative to β-actin.

QPCR reactions were performed using SYBR Premix Ex Roche (Roche SYBR Green I, Swit.) in a total volume of 20 μl containing 1 µl of cDNA, 0.5 µl of forward and 0.5 µl of reverse primers. The qPCR reactions were performed as follows: initial denaturation at 95 °C for 10 min, amplification (95 °C for 30 s, 55 °C for 30 s, 72 °C for 30 s, 33 cycles). QPCR with GAPDH-specific primers was run in parallel in each sample as an internal control. The primers for AKR1B10 were 5′-CAG AAG ACC CTT CCC TGC TG (forward) and 5′-CGT TAC AGG CCC TCC AGT TT (reverse), and for GAPDH were 5′-GAA GGT GAA GGT CGG AGT CA (forward) and 5′-GGA AGA TGG TGA TGG GAT TTC (reverse). The relative amount of AKR1B10 mRNA to GAPDH was calculated as 2^∆CT^, where ∆CT = Ct_AKR1B10_ − Ct_GAPDH_.

### Cell culture and stable clone establishment

Human nasopharyngeal carcinoma (hNPC) CNE-2 cells were obtained from the Xiangya Hospital, Changsha, China, were grown in RPMI-1640 medium (Hyclone, UT) supplemented with 10 % fetal bovine serum (Gibco/BRL, Grand Island, NY) and 100 U/ml penicillin and 100 mg/ml streptomycin at 37 °C in a 5 % CO_2_ atmosphere. To overexpress AKR1B10 in CNE-2 cells, we cloned AKR1B10 gene into a modified lentivirus vector in which AKR1B10 was co-expressed with puromycine-resistant gene (pSIN-EF2-AKR1B10-Puromycin). The lentivirus vector pSIN-EF2-AKR1B10-Puromycin or control empty lentivirus vector pSIN-EF2-Puromycin together with auxiliary plasmids pSPAX2 and pMD2.G were transfected into 293T cells to package lentivirus. After infected by lentivirus, CNE-2/AKR1B10 cell clones and the control CNE-2/vector cells were screened out through adding puromycin into the mediums. The stable expression of AKR1B10 was detected using western blot method. CNE-2/AKR1B10 and CNE-2/vector cells were used in the following experiments.

### Protein extraction and western blot

Total cellular proteins were extracted always on ice using Roche lysate buffer (Roche, Switzerland). Protein concentration was quantitated with BCA method. 45 μg of total protein extracts were loaded in 12 % SDS-PAGE gel, electrophoresed at 80–120 V at room temperature, then transferred to a nitrocellulose membrane (Millipore, Bedford, MA) at 200 mA for 90 min at 4 °C. After blocked with 5 % milk in PBST buffer for 45 min at the room temperature, the membrane was incubated with self-prepared rabbit anti-AKR1B10 primary antibody (1:500) and mouse anti-β-actin antibody (1:5000) (Sigma-Aldrich Company, USA) for 8 h at 4°, and then incubated with secondary antibodies tagged with chemoluminescence for 1 h at room temperature. Finally the protein expression levels in the membrane were detected by Titative Analysis System (Alpha Fluor chem Q, protein simple, USA).

### MTT assay

CNE-2/vector and CNE-2/AKR1B10 cells (5 × 10^3^ or 7 × 10^3^ per well) were seeded into 96-well plates and cultured for 72 h. 20 µl of 3-(4,5-dimethylthiazol-2-yl)-2,5-diphenyl tetrazolium bromide (MTT) were added into each well. The cells were incubated at 37 °C for 4 h. Subsequently, the supernatant in each well was discarded, and 150 µl of dimethylsulfoxide were then added into each well. The plates were shaken for 10 min to make the crystals fully dissolved. Finally, the OD value was measured at 490 nm with the multiscan spectrometer (Thermo Varioskan Flash, Wilmington, DE) and the percentage of viable cells of CNE-2/AKR1B10 normalized to CNE-2/vector was calculated.

### Cell cycle assay

CNE-2/vector and CNE-2/AKR1B10 cells (1 × 10^6^ in a 60 mm-diameter dish) were incubated in medium with 10 % FBS for 24 h. After collected and fixed in 750 µl ice-cold alcohol for 2 h at 4 °C. The fixed cells were collected by centrifuging at 1000*g* for 5 min. washing with 1 ml ice-cold PBS and then resuspended in 250 µl ice-cold PBS, incubated in PBS containing 10 µl RNase A for 30 min at 37 °C. Finally, 12 µl of propidium iodide (PI) solution were added to each tube for 30 min on ice. The samples were immediately examined with red fluorescence at a wavelength of 488 nm by using a FACstar Plus cytometer (Beckman Germany). Light scattering was detected at the same time. The cellular DNA content and light scattering were analyzed by Cell Quest software (Becton–Dickinson, USA). PIs [(cell number in S + G2/M phages)/(total cell number)] were calculated to identify cell proliferation.

### Scratch assay

The same number of CNE-2/vector and CNE-2/AKR1B10 cells was cultured in a 24-well plate. When the cells were cultured to confluency, they were then scratched with a 200 μl pipette tip and further incubated in DMEM supplemented with 1 % FBS. Images were taken at 24, and 48 h with a Leica light microscope DM4 B (Leica Corporation, Germany). The Image-Pro plus image analysis system was used to measure the lesion area. The data were expressed as cell migration ratio (MR %) using the equation: MR % = [(Area at T_0_ − Area at T_t_)/Area at T_0_,] × 100 %, where T_t_ is the number of hours post-injury and T_0_ is the time of injury.

### Statistical analysis

Statistical analysis was performed using the Statistical Package for Social Sciences (SPSS) for Windows, version 18.0 (SPSS, Chicago, IL, USA). All data were described as mean ± SD. Fisher’s exact test and Spearman correlation analysis were produced to examine the relationship between AKR1B10 expression and clinicopathological variables. *T* test was used to analyze cell proliferation and cell migration. Results were considered statistically significant for *p* < 0.05.

## Abbreviations

AKR1B10: Aldo–keto reductase B10
NPC: nasopharyngeal carcinoma
AKRs: aldehyde ketone reductase
EBV: Epstein-Barr virus
MMP-1: matrix metalloproteinase 1
TFPI-2: tissue factor pathway inhibitory factor 2
ARL-1: aldose reductase like protein-1
qRT-PCR: quantitative reverse transcription polymerase chain reaction
